# Molecular determinants and interaction data of cyclic peptide inhibitor with the extracellular domain of TrkB receptor

**DOI:** 10.1016/j.dib.2016.01.016

**Published:** 2016-01-16

**Authors:** Nitin Chitranshi, Vivek Gupta, Yogita Dheer, Veer Gupta, Roshana Vander Wall, Stuart Graham

**Affiliations:** a Faculty of Medicine and Health Sciences, Macquarie University, F10A, 2 Technology Place, North Ryde, NSW 2109, Australia; b School of Medical Sciences, Edith Cowan University, Perth, Australia; c Save Sight Institute, Sydney University, Sydney, NSW 2109, Australia

**Keywords:** TrkB, Cyclotraxin B, GSK3β, Docking

## Abstract

TrkB is a high affinity receptor for the brain derived neurotrophic factor (BDNF) and its phosphorylation stimulates activation of several intracellular signalling pathways linked to cellular growth, differentiation and maintenance. Identification of various activators and inhibitors of the TrkB receptor and greater understanding their binding mechanisms is critical to elucidate the biochemical and pharmacological pathways and analyse various protein crystallization studies. The data presented here is related to the research article entitled “Brain Derived neurotrophic factor is involved in the regulation of glycogen synthase kinase 3β (GSK3β) signalling” [1]. Cyclotraxin B (CTXB) is a disulphide bridge linked cyclic peptide molecule that interacts with TrkB receptor and inhibits the BDNF/TrkB downstream signalling. This article reports for the first time binding mechanism and interaction parameters of CTXB with the TrkB receptor. The molecular model of CTXB has been generated and it’s docking with TrkB domain carried out to determine the critical residues involved in the protein peptide interaction.

**Specifications Table**

**Table:** 

Subject area	*Biology*
More specific subject area	*Protein-peptide binding and interactions*
Type of data	*Table, protein-peptide interaction images and tables*
How data was acquired	*Molecular modeling and in silico analysis*
Data format	*Analysed*
Experimental factors	*Molecular modeling and docking*
Experimental features	*Amino acid orientations and type of interactions, energy minimization.*
Data source location	*Australia*
Data accessibility	*Data within this article*

**Value of the data**

• Three dimensional molecular model of the cyclic peptide cyclotraxin B was generated for the first time to determine its interactions with the TrkB receptor.
• Future studies to determine pharmacological, biochemical or interaction studies of TrkB receptor or related molecules will be facilitated by the interaction and binding parameters reported here.
• Binding interactions of CTXB with TrkB will determine its potential usage in pharmacological studies and development of new derivatives and ligands.

## 1 Data

The data shown here elucidates molecular modeling of cyclic peptide cyclotraxin B which is a TrkB inhibitor. Active site of the extracellular D5 domain of the TrkB receptor which is primarily involved in this interaction was also modelled. Finally the cyclic peptide inhibitor was also identified and docking carried out with the extracellular D5 domain of TrkB. The interacting amino acid residues that are involved in docking are identified and various interaction parameters provided in detail using a combination of molecular modeling and molecular docking computational tools.

## 2 Experimental design, materials and methods

### 2.1 Selection and preparation of cyclotraxin B, TrkB inhibitor

**Fig. 1:**
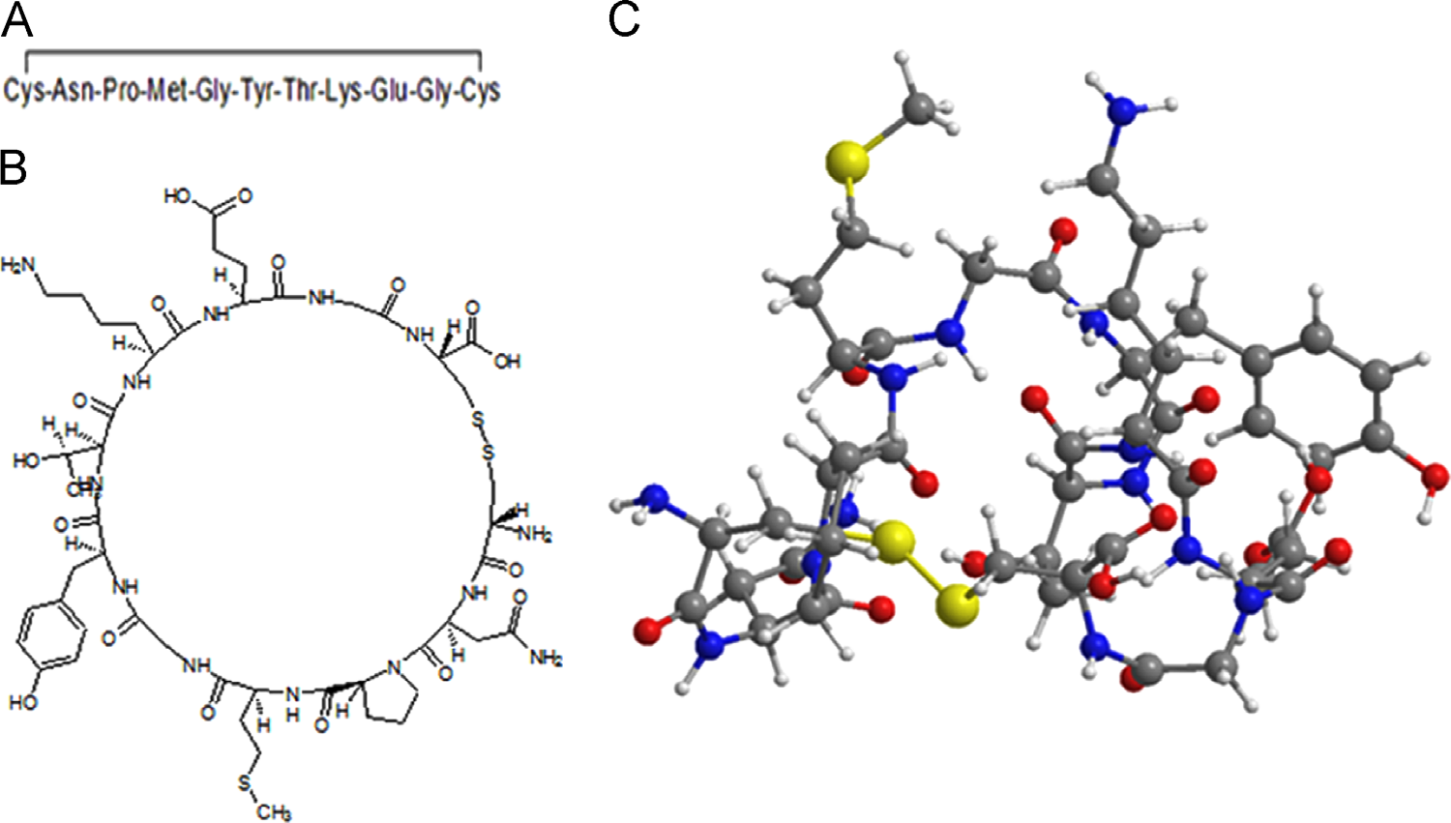
Cyclotraxin B stuructre (A) one dimensional, (B) two dimensional and (C) three dimensional view.

Cyclotraxin B (CTXB) is a cyclic peptide chain of 10 amino acid linked by a disulphide bridge [2]. It is an inhibitor of the TrkB receptor activation and its downstream signalling pathway mediated by BDNF binding [1], [3], [4], [5]. Cyclization is important to provide stability to the peptide macromolecule. The primary structure of the peptide is known and in this manuscript we report for the first time the putative three dimensional structure of the peptide (Fig. 1A). The two dimensional (2D) and three dimensional (3D) structure of the CTXB was built using ChemDraw Ultra 8.0 (Cambridgesoft, Waltham, MA, USA) (Fig. 1B and C). Extensive energy minimization was performed using the Austin Model-1 (AM1) programme until the root mean square (RMS) gradient value became smaller than 0. 100 kcal/mol Å. The molecule was further subjected to re-optimization *via* MOPAC (Molecular Orbital Package) method [6] until the RMS gradient attained a value lesser than 0.0001 kcal/mol Å. The chemical properties of CTXB was calculated by ACD (Advanced Chemistry Development, Canada) labs Chemsketch software and the data is presented in Table 1 [7].

### 2.2 Molecular modeling and generation of TrkB binding region

The primary structure of TrkB receptor and its various domains were examined [8]. Crystal structure of the extracellular D5 domain of the TrkB which exhibits binding with the human Neurotrophin-4/5 (NT-4/5) ligand (PDB id: 1HCF) was selected from the protein databank [9]. The asymmetric unit of the crystal structure contains a single copy of the TrkB-D5:NT-4/5 complex, comprising one homodimer of NT-4/5 bound to two monomers of TrkB-D5. The two monomer chain of TrkB-D5 (chains X and Y) are identical. These chains are known to interact with the BDNF/ NT-4/5 protein. Only chain X of PDB id 1HCF was considered in the present study for its potential interactions with the CTXB that highlights the location of the conserved interaction site. The optimization of proteins was carried out using well characterized UCSF Chimera software (San Francisco, California, USA), implying amber parameters, followed by minimization with MMTK (Molecular Modeling Toolkit) method. The steepest minimizations was first performed in 1000 steps to relieve highly unfavourable clashes than followed by conjugate gradient minimization in 500 steps with a step size of 0.02 Å for more effective reaching an energy minimum [10], [11].

### 2.3 Identification of CTXB binding site

The protein motif was subjected to *in silico* assessment of the potential binding of selected CTXB residues to different regions on the TrkB D5 surfaces. This binding interactions were assessed in terms of probability of CTXB participating residues binding to the TrkB D5 receptor surface and theoretical scores determined using the PepSite2 server [12]. Peptide binding may also affect the tyrosine phosphorylation profile [13] of the TrkB receptor translating into its altered activity which can be assessed in future investigations. Surface accessibility of the ligand to various binding pockets was also examined [14].

**Fig. 2:**
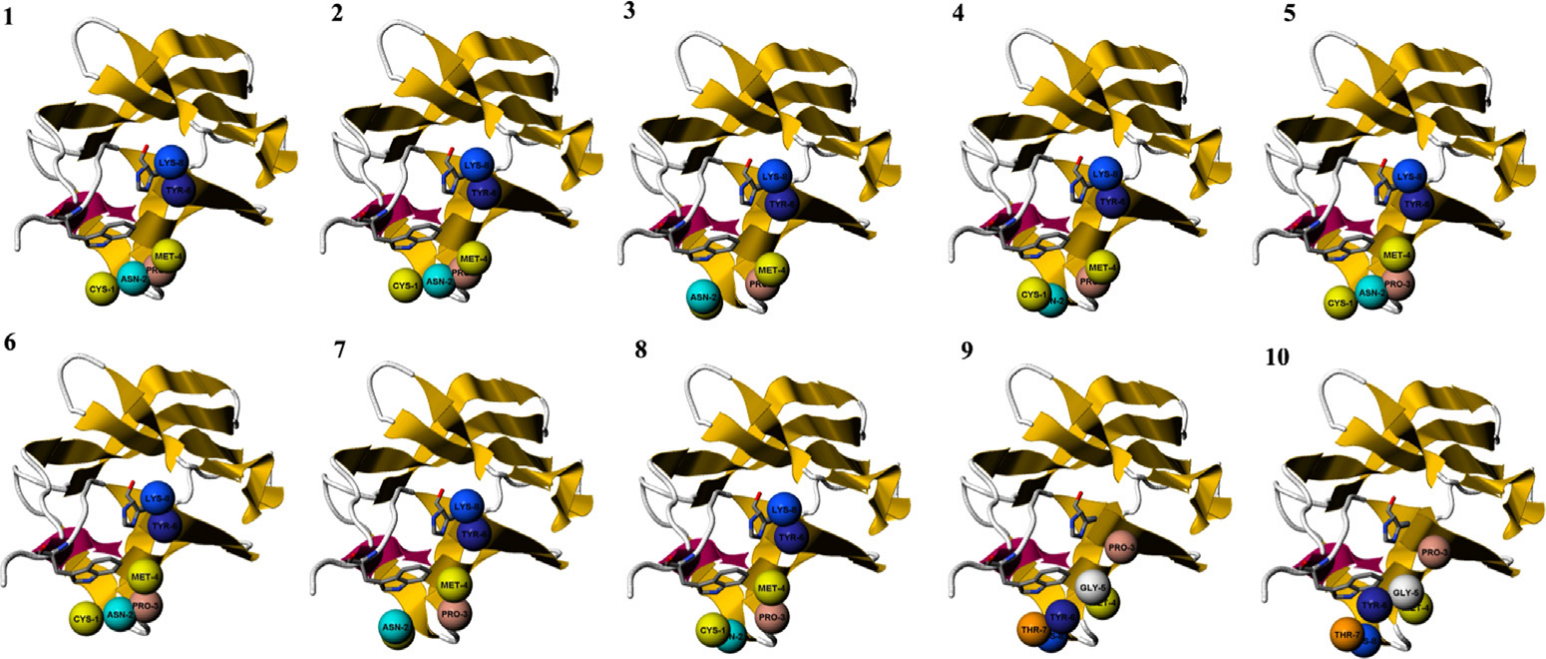
Predicted binding sites of CTXB peptide ‘‘CNPMGYTKEG’’ core motif on the surface of TrkB-D5 domain as predicted by the PEPSITE2 programme. The six ball shaped structures indicate the predicted locations of six residues from ‘‘CNPMGYTKEG’’. (1–8) distribution of 6 amino acids of the peptide predicted on the surface of TrkB-D5 in the sequence of Cys (C), Asn (N), Pro (P), Met (M), Tyr (Y) and Lys (K) (labelled) and (9–10) distribution of 6 selected amino acids of CTXB binding to TrkB-D5-Pro (P), Met (M), Gly (G), Tyr (Y), Thr (T) and Lys (K) (labelled).

PepSite2 is a computational tool that scans the surface of a given protein for patches or grooves that are likely to influence binding of individual amino acid residues or peptides up to ten amino acids and provides a score that reflects the propensity of the peptide to bind to that region. The PepSite score is expressed in relative units and the higher scores reflects superior binding. We applied PepSite in a sliding window of 10 residues to assess the binding of the TrkB-D5 domain and CTXB peptide sequence (CNPMGYTKEG). The scores of the CTXB binding to different regions of TrkB D5 domain are presented (Fig. 2A, Model 1, Table 2) [1].

### 2.4 Peptide docking in the binding region

**Fig. 3:**
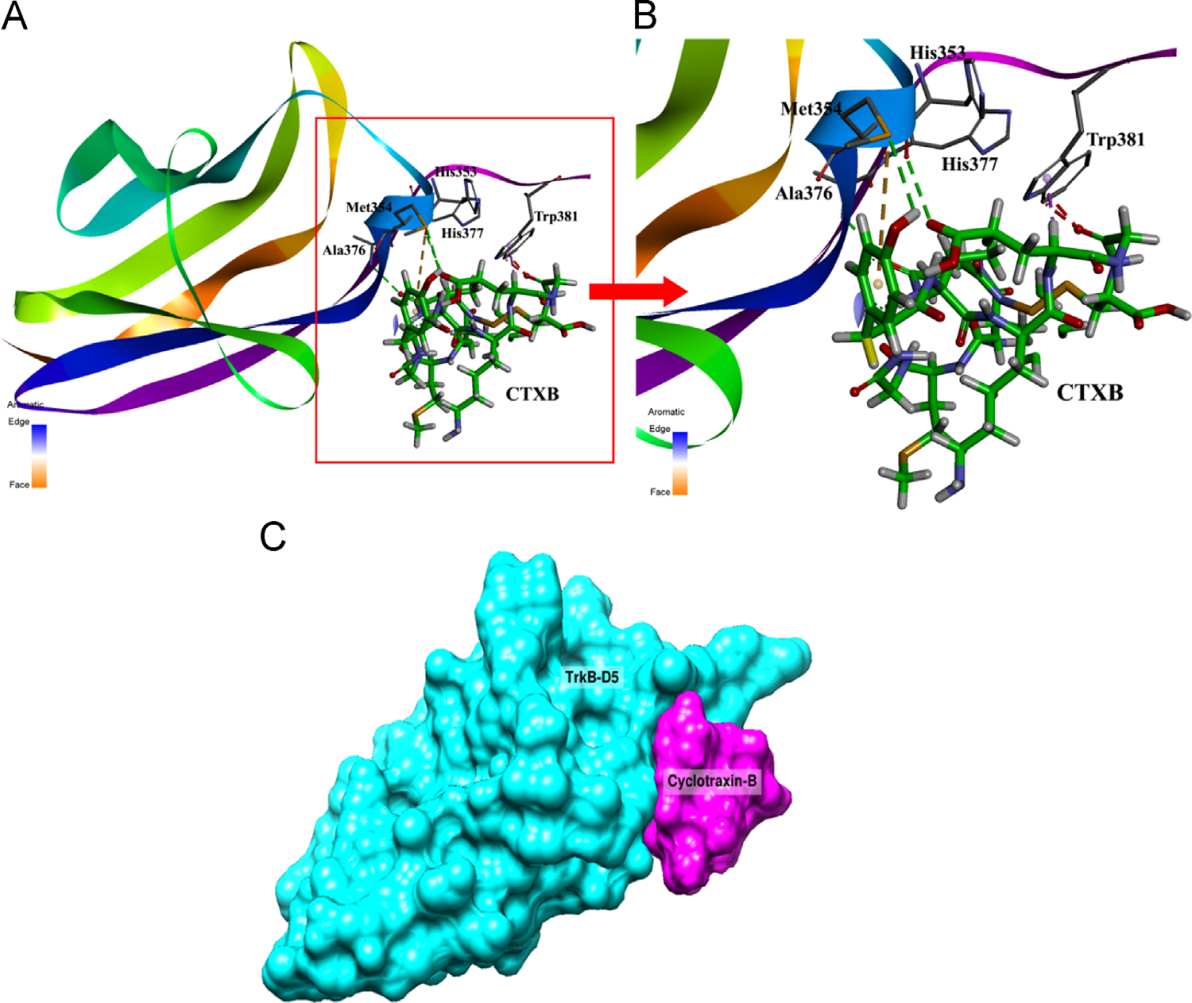
Interacting residues and binding mode of CTXB with TrkB-D5 domain (A) Docking of TrkB (ribbon structure) with the CTXB (stick model) showing critical residues (rank 1) involved in interaction, (B) enlarged view of the interaction pocket within 5.5 Å region around the ligand, CTXB-TrkB-D5 complex and (C) surface view showing the grove in TrkB-D5 domain (Cyan) locating CTXB peptide (pink). Green dashed line denotes the hydrogen bonding and brown dashed line reflects pi-sulpha interactions and stacking. The images were generated with the Discovery Studio 4.0 Client (Accelrys, Inc., San Diego, CA, USA).

The protein-peptide docking was performed with the PatchDock software available in the public domain [15]. The crystal structure of TrkB (PDB ID: 1HCF) was retrieved from Protein Data Bank. The single chain of TrkB-D5 as described previously was used for docking study under default complex-type settings. Molecular visualization and general analysis were done using the program PyMOL and Discovery Studio 4.0 softwares [16]. TrkB residues closer than 5.5 Å to any of the C-terminal NT-4/5 amino acids (Cys345-Asn350) were selected for docking (Fig. S1). The docking scores, atomic contact energies and geometrical parameters are compiled as Table 3. The hydrogen bond between TrkB-D5 and CTXB (rank 1) was observed to be formed between the amino acid residues His353, Met354, Ala376, His377 and Trp381 (Fig. 3A). The pi-sulpha interaction was also observed between CTXB and Met354 (Fig. 3B). Surface binding of CTXB with TrkB-D5 domain is shown in Fig. 3C. The selected binding pocket of TrkB-D5 was observed to overlap with the C-terminal region of the NT-4/5 binding site (Fig. S2). The two dimensional (2D) interaction map was created by LigPlot+ which helped to identify new interacting residues in the TrKB-D5:CTXB complex [17]. Ser294, Asn320, Gln373 and Ser375 of TrkB-D5 were identified to form hydrogen bonds with CTXB. Trp381 was observed to be involved in CTXB TrkBD5 binding using both the Discovery Studio 4.0 as well as the LigPlot+ programmes (Fig. S3).

## Figures and Tables

**Table 1:** The chemical property and the calculation of cyclotraxin B (CTXB) as evaluated using ACD labs Chemsketch software.

**Chemical properties**	**Calculations**
Molecular formula	C_48_H_73_N_13_O_17_S_3_
Formula weight	1200.36512
Composition	C(48.03%) H(6.13%) N(15.17%) O(22.66%) S(8.01%)
Molar refractivity	296.25±0.4 cm3
Molar volume	805.8±5.0 cm3
Parachor	2467.9±6.0 cm3
Index of refraction	1.656±0.03
Surface tension	87.9±5.0 dyne/cm
Density	1.48±0.1 g/cm3
Dielectric constant	Not available
Polarizability	117.44±0.5 10-24 cm3
Monoisotopic mass	1199.440948 Da
Nominal mass	1199 Da
Average mass	1200.3651 Da

**Table 2:** PepSite2 binding score prediction of selected cyclotraxin B (CTXB) residue sequences to the TrkB-D5 domain.

**Rank**	**Peptide sequence order**	**Pepsite2 score**
1	CNPMYK	0.02733
2	CNPMYK	0.03015
3	CNPMYK	0.03266
4	CNPMYK	0.03583
5	CNPMYK	0.03719
6	CNPMYK	0.04098
7	CNPMYK	0.04435
8	CNPMYK	0.04993
9	PMGYTK	0.05985
10	PMGYTK	0.06992

**Table 3:** The top 20 docking scores and geometrical parameters of the peptide CTXB with TrkB-D5 domain; ACE: Atomic Contact Energy.

**Rank**	**Score**	**Area**	**ACE**	**Transformation**

1	5538	652.1	−344.82	−0.57 −1.01 −2.62 −10.48 −3.78 29.57
2	5282	597.6	−272.85	−1.01 −0.26 0.32 −12.66 −4.68 28.84
3	5100	620	−330.86	–1.52 0.07 0.81 –11.83 –7.13 27.44
4	4966	528.9	−126.23	–0.31 –0.63 –2.16 –22.34 8.83 21.59
5	4878	623.4	−300.47	2.71 0.78 −1.27 −13.25 −4.12 29.20
6	4824	571.7	−214.66	–0.11 1.20 2.50 –5.75 11.23 7.03
7	4800	621.7	−217.57	–2.59 –0.46 0.54 –22.14 6.07 23.60
8	4678	531.9	−182.76	−0.67 −0.16 −1.87 −20.33 9.72 23.42
9	4654	512.8	−234.35	0.84 −0.57 0.51 −12.13 −2.05 31.89
10	4642	570.9	−250.43	−1.34 0.11 −1.76 −23.69 10.32 20.31
11	4606	539.9	−170.59	0.32 −1.01 2.51 −22.65 10.28 21.86
12	4536	521.6	−189.48	−3.07 −0.42 0.56 −20.13 9.32 24.74
13	4530	735.8	−436.61	−0.46 1.21 −2.78 −3.58 11.15 12.99
14	4522	622	−273.37	−1.99 −0.45 2.81 −8.24 −1.98 30.78
15	4358	547.6	−276.07	0.05 −0.96 2.97 −7.11 −6.08 31.48
16	4264	468.6	−196.84	2.26 –0.23 2.23 –1.86 14.82 14.52
17	4242	531.7	−154.98	−0.67 –0.38 −2.13 −16.81 14.23 21.56
18	4240	548.7	−304.69	0.50 −0.35 −0.24 −6.28 11.67 5.72
19	4234	458.8	−155.03	2.26 −0.60 −2.85 −2.98 13.89 13.16
20	4230	653.7	−351.22	−0.76 1.23 2.83 −5.51 13.82 10.66

## Footnotes

Appendix A Supplementary data associated with this article can be found in the online version at doi:10.1016/j.dib.2016.01.016.
