# Supplementary material for: Molecular determinants and interaction data of cyclic peptide inhibitor with the extracellular domain of TrkB receptor
Source: Data Brief. 2016 Jan 16;6:776–82. doi: 10.1016/j.dib.2016.01.016 (PMC4744334; doi:10.1016/j.dib.2016.01.016)
Supplement: Supplementary file 2 — Supplementary material [file mmc2.docx]

*Data article*

**Title: Molecular determinants and interaction data of cyclic peptide inhibitor with the extracellular domain of TrkB receptor**

Authors: Nitin Chitranshi ^1,†,^*, Vivek Gupta ^1,†^, Yogita Dheer ^1^, Veer Gupta ^2^ , Roshana Vander Wall ^1^, Stuart Graham ^1,3^

**Affiliations:**

^1^ Faculty of Medicine and Health Sciences, Macquarie University, F10A, 2 Technology Place,
North Ryde, NSW 2109, Australia;

^2^ School of Medical Sciences, Edith Cowan University, Perth, Australia

^3^ Save Sight Institute, Sydney University, Sydney NSW 2109, Australia

**^†^** These authors contributed equally to this work.

**Contact email:**

[nitinchitranshi@gmail.com](mailto:nitinchitranshi@gmail.com) (Nitin Chitranshi), [vivek.gupta@mq.edu.au](mailto:vivek.gupta@mq.edu.au) (Vivek Gupta)


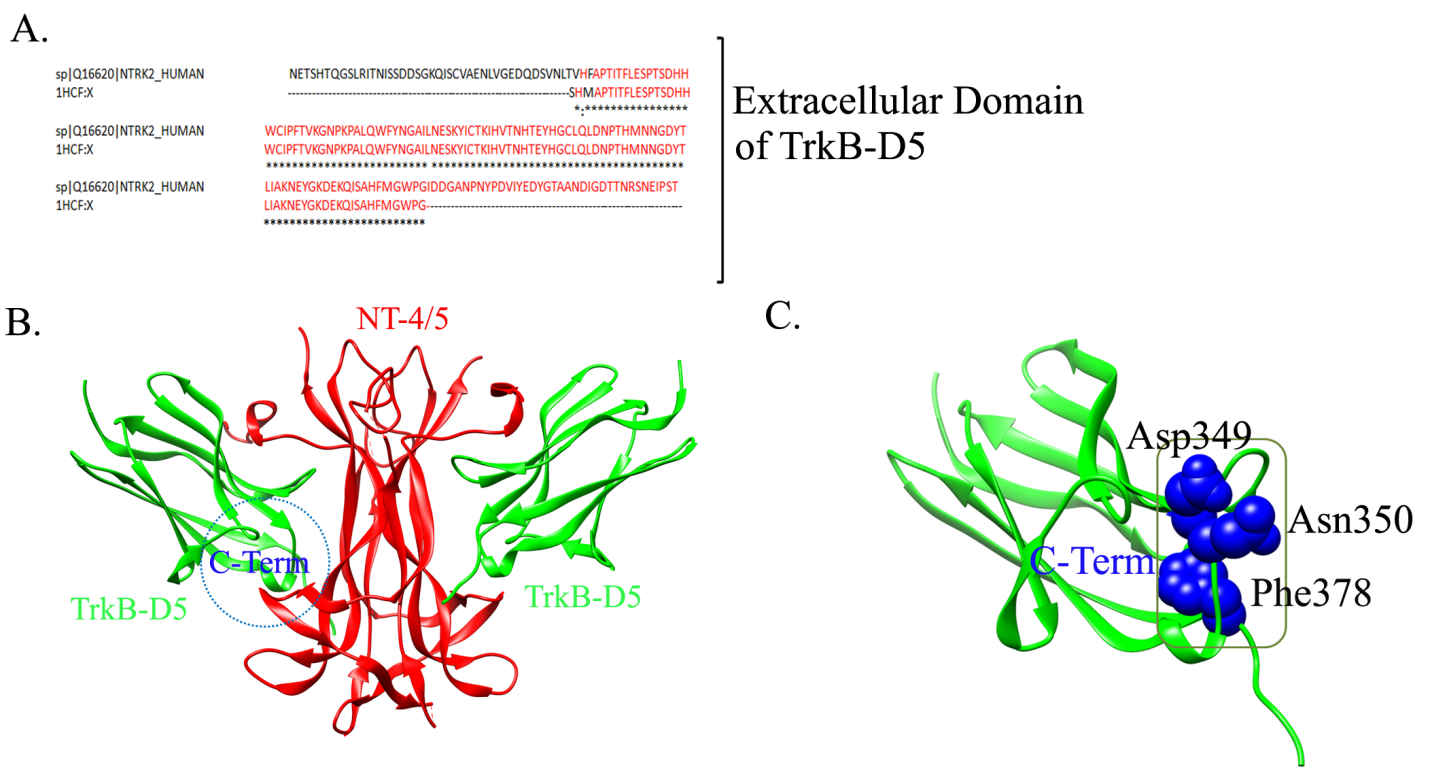


**Figure S1.** Schematic representation of extracellular domain (ECD) of TrkB-D5 at region C-terminal (C-Term) patch created using computational modeling. (A) An alignment of the ECD amino acid sequence of human TrkB and X chain of PDB: 1HCF (the aligned amino acids are colored red and stared; dashes represent gaps). (B) The 3D model of the complex between the TrkB-D5 domain (green) and the NT-4/5 (red) along with the C-Term region selected for docking site. (C) Molecular docking was performed targeting the specificity path at C-Term segment of TrkB-D5 (green; Asp349, Asn350 and Phe378).


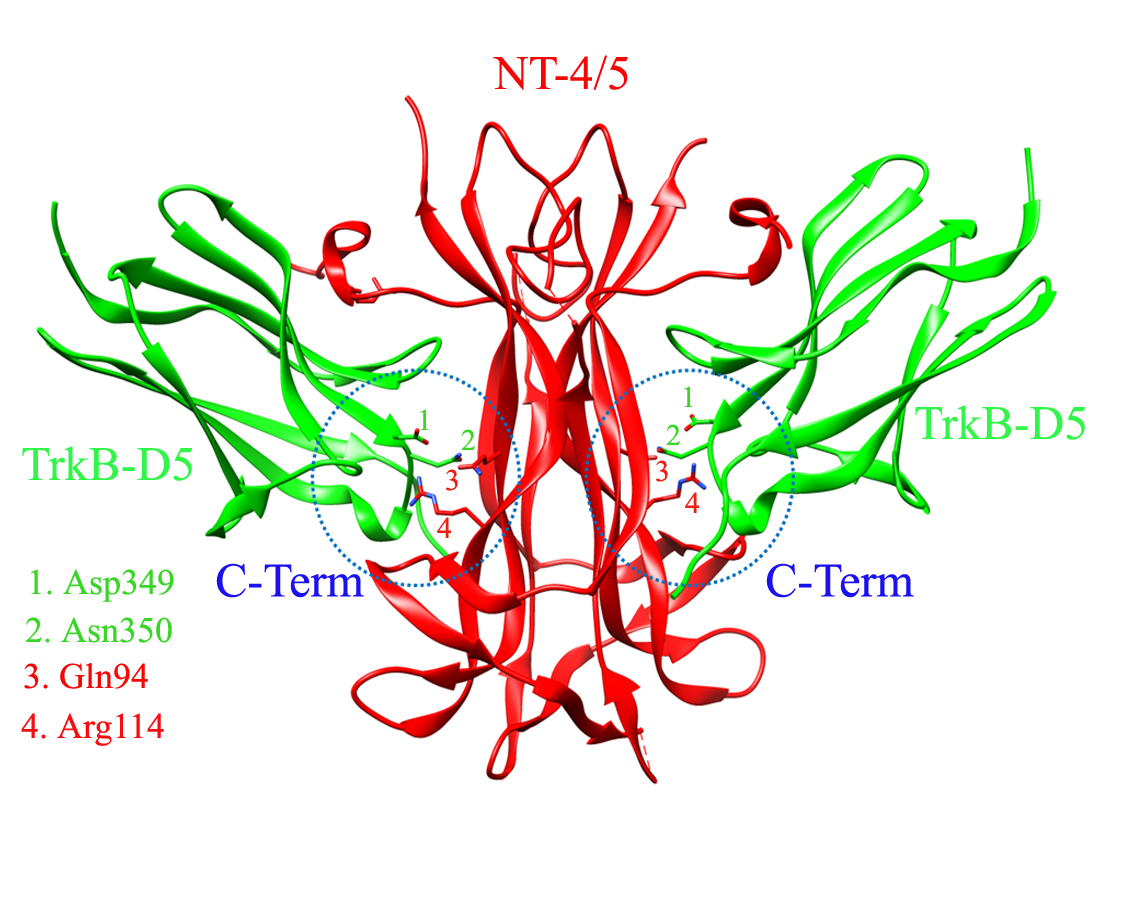
**Figure S2.** Schematic representation of TrkB-D5:NT-4/5 complex at C-terminal (C-Term) patch. The 3D model of the complex between the TrkB-D5 domain (green) and the NT-4/5 (red) was identified that overlap with the residues of TrkB-D5:NT-4/5 complex. Molecular docking was performed targeting the specificity path at C-Term segment of TrkB-D5 (green; Asp349 and Asn350) and NT-4/5 (red; Gln94 and Arg114).

**Figure S3.**
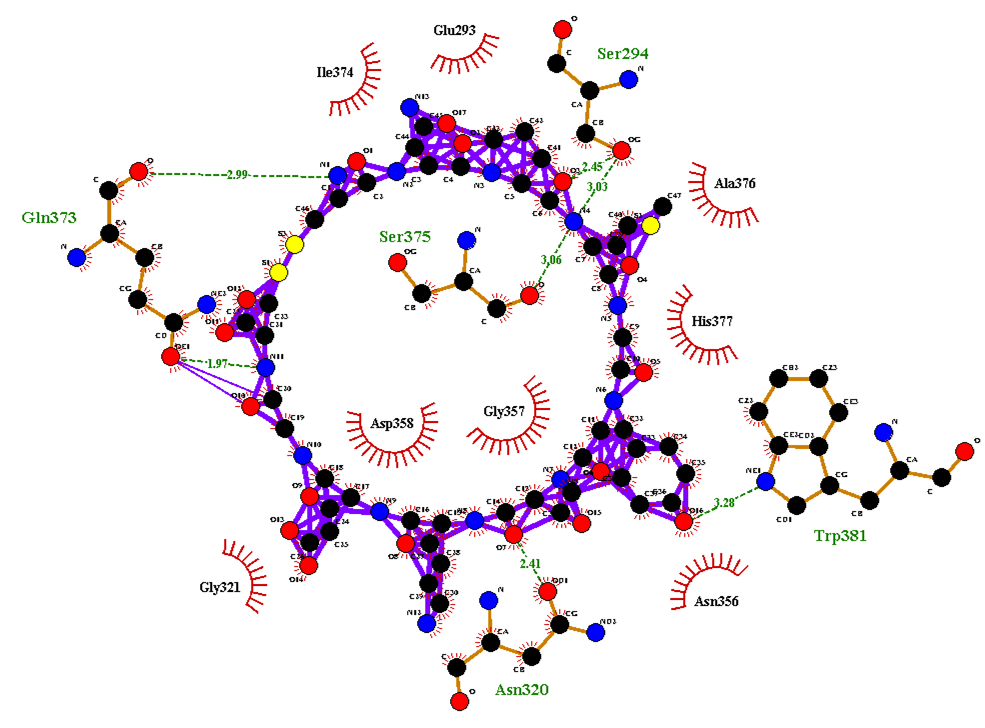
2D interaction map obtained from LigPlot. Green dashed line represents the hydrogen bond between TrkB-D5 and CTXB. Red color represents the hydrophobic residues of TrkB-D5 surrounding CTXB. TrkB antagonist (CTXB) is represented in purple color.
